# Supplementary material for: In Vitro Antibacterial Activity of Ozonated Olive Oil against Bacteria of Various Antimicrobial Resistance Profiles Isolated from Wounds of Companion Animals
Source: Int J Mol Sci. 2024 Mar 21;25(6):3557. doi: 10.3390/ijms25063557 (PMC10971217; doi:10.3390/ijms25063557)
Supplement: Supplementary file 1 [file ijms-25-03557-s001.zip › ijms-2915653-supplementary.pdf]

Table S1. List of bacteria species used in the experiment.

Species	Total (n=101)
Gram-negative	44 (43.56)
Acinetobacter calcoaceticus	1
Acinetobacter pittii	1
Acinetobacter ursingii	3
Aeromonas media	1
Brevundimonas diminuta	1
Citrobacter freundi	2
Enterobacter hormaechei	2
Escherichia coli	11
Klebsiella pneumoniae	1
Kocuria rhizophilia	1
Leclercia adecarboxylata	1
Pantoea agglomerans	2
Pantoea sp.	1
Moraxella osloensis	1
Proteus mirabilis	2
Proteus vulgaris	1
Pseudomonas aeruginosa	1
Pseudomonas putida	2
Pseudomonas graminis	1
Pseudomonas fulva	1
Psychrobacter sanguinis	2
Psychrobacter pulmonis	1
Serratia marcescens	1
Serratia liquefaciens	1
Stenotrophomonas maltophilia	1
Gram-positive	57 (56.44)
Bacillus pumilus	1
Enterococcus avium	1
Enterococcus faecalis	9
Enterococcus faecium	3
Enterococcus hirae	2
Lysinibacillus fusiformis	1
Macrococcus canis	1
Micrococcus luteus	1
Microbacterium maritypicum	1
Microbacterium oxydans	1
Microbacterium paraoxydans	1
Peribacillus simplex	1
Sporosarcina luteola	1
Staphylococcus aureus	4
Staphylococcus capitis	1
Staphylococcus cohnii	1
Staphylococcus devriesei	1
Staphylococcus condimenti	1
Staphylococcus felis	1
Staphylococcus haemolyticus	1
Staphylococcus pasteuri	1
Staphylococcus lentus	1
Staphylococcus pseudintermedius	12

Staphylococcus schleiferi	2
Staphylococcus saprophyticus	1
Staphylococcus warneri	1
Staphylococcus sciuri	1
Streptococcus canis	4
